# Comparative Molecular Docking Analysis of Cytoplasmic Dynein Light Chain DYNLL1 with Pilin to Explore the Molecular Mechanism of Pathogenesis Caused by *Pseudomonas aeruginosa* PAO

**DOI:** 10.1371/journal.pone.0076730

**Published:** 2013-10-03

**Authors:** Samina Kausar, Muhammad Asif, Nousheen Bibi, Sajid Rashid

**Affiliations:** National Center for Bioinformatics, Quaid-i-Azam University, Islamabad, Pakistan; University of South Florida College of Medicine, United States of America

## Abstract

Cytoplasmic dynein light chain 1 (DYNLL1) is a component of large protein complex, which is implicated in cargo transport processes, and is known to interact with many cellular and viral proteins through its short consensus motif (K/R)XTQT. Still, it remains to be explored that bacterial proteins also exhibit similar recognition sequences to make them vulnerable to host defense mechanism. We employed multiple docking protocols including AUTODOCK, PatchDock, ZDOCK, DOCK/PIERR and CLUSPRO to explore the DYNLL1 and Pilin interaction followed by molecular dynamics simulation assays. Subsequent structural comparison of the predicted binding site for DYNLL1-Pilin complex against the experimentally verified DYNLL1 binding partners was performed to cross check the residual contributions and to determine the binding mode. On the basis of *in silico* analysis, here we describe a novel interaction of DYNLL1 and receptor binding domain of Pilin (the main protein constituent of bacterial type IV Pili) of gram negative bacteria *Pseudomonas aeruginosa* (PAO), which is the third most common nosocomial pathogen associated with the life-threatening infections. Evidently, our results underscore that Pilin specific motif (KSTQD) exhibits a close structural similarity to that of Vaccinia virus polymerase, P protein Rabies and P protein Mokola viruses. We speculate that binding of DYNLL1 to Pilin may trigger an uncontrolled inflammatory response of the host immune system during *P. aeruginosa* chronic infections thereby opening a new pioneering area to investigate the role of DYNLL1 in gram negative bacterial infections other than viral infections. Moreover, by manifesting a strict correspondence between sequence and function, our study anticipates a novel drug target site to control the complications caused by *P. aeruginosa* infections.

## Introduction

Cytoplasmic dynein is a part of large protein complex which functions as a minus-end directed microtubule based motor with the intermediate and light chains, doubtlessly entangled in the dynein binding to the appropriate cargo transport processes [1]–[2]. Among dynein light chains, human 8 kDa dynein light chain type 1 (DYNLL1) which was initially elucidated as a subunit of *Chlamydomonas* axonemal dynein [3], prevails in multiple functions. This light chain is found to be highly similar to a subset of mammal, nematode, insect, metazoan, plant, bacteria and yeast [4].

In recent years, DYNLL1 has been reported to bind multiple target proteins including GRINL1A, p54, postsynaptic protein (gephyrin), neuronal nitric oxide synthase (nNOS), DIC, Bim, EML3, p21-activated kinase and Swallow [5]–[7]. Frequently, the short linear DYNLL1 interacting motifs are positioned in natively disordered protein segments [8] and exist near to the coiled-coil or other dimerization domains of the binding partners. DYNLL1 binding motifs are originally divided into two classes: K/R-XTQT or (K)3 X)2 T)1 Q0 T1 X2) and G-I/V-QVD or [X)3 G)2 (I/V))1 Q0 V1 D2] [7]–[9]. The central Gln (at position 0) in both classes caps the N-terminal end of the second alpha-helix, while residues at positions +1, 1 and 3 bind with the endogenous region of the binding groove. Some DYNLL1 interacting partners consist of unorthodox binding motifs, which lack the highly conserved Gln residue e.g. Pak1 [10] GRINL1A [5] and MYO5A [11], [12]. However, the general interacting topology of these peptides is similar to that of the recognized ones. Accordingly, a specific H-bond network balances the lack of conserved Gln [10] in the DYNLL1/Pak1 complex, and in the docking model of DYNLL1/GRINL1A [5].

DYNLL1 contains an exclusive fold with two five-stranded antiparallel beta-sheets, which are accountable for dimerization, while every beta-sheet consists of four strands originated from one monomer and a fifth strand comes from the other monomer. These beta-sheets are enclosed by two pairs of alpha-helices [13], [14]. The attached ligands of DYNLL1 lie in the two parallel grooves formed at the two brinks of the dimerization interface and form an additional antiparallel beta-strand thereby enlarging the central beta-sheets. Almost all dissimilar residues of DYNLL1 paralogs and orthologs in metazoans lie at the outer surface of the homodimer protein which lack binding contribution [7].

In human, since DYNLL1 homodimer has been reported to bind with KETQTP motif located in the intermediate chain, where it adopts an extended conformation, it is generally considered that most of the interacting cellular and viral peptides exhibit similar recognition sequences and interact in a similar fashion [15]. For intracellular transport, viruses use two approaches; either hijack the cytoplasmic membrane traffic or interact directly with the cytoskeletal transport machinery [9]. Upon infection, several viruses exploit the cellular cytoplasmic transport machinery to allow virions or subviral nucleoprotein complexes to travel long distances through the cytoplasm from the cell surface to the site of viral transcription and replication. Because free diffusion of molecules (larger than 50 nm in diameter) is restricted by the structural organization of cytoplasm, viruses as well as cellular organelles require active transport mechanism along microtubules [9], [15]. This is specifically obvious in case of neurotropic viruses which move long distances during retrograde or anterograde transport in the axon [9].

Moreover, integrity of the microtubules is interconnected and essential for viral infection, as demonstrated by experiments in which microtubule-depolymerizing agents such as colchicine,vinblastine or nocodazole were used. Such pathogens causing widespread illness include adenovirus [16], [17], parvovirus [18], herpes simplex virus (HSV) [16], [19], human cytomegalovirus [20], hepatitis B virus [21], african swine fever virus (ASFV) [22], rabies virus (RV) [23], influenza virus [24], human immunodeficiency virus (HIV) [25] and papillomavirus (PV) [26]. These viruses depend on the host microtubules for persuasive nuclear targeting and efficacious infection, a common mechanism for their delivery alongside the cell nucleus replication site [15].

In this study, we reported a novel infection model for gram negative bacteria PAO (a strain of *Pseudomonas aeruginosa*) based on a direct interaction of Fimbrial protein Pilin and DYNLL1. *P. aeruginosa* is a successful opportunistic bacteria being the third most common nosocomial pathogen in the world associated with life-threatening infections in the setting of epithelial injury, in part due to its ability to colonize a wide spectrum of living and nonliving surfaces using its type IV Pili, which are the polar flexible filaments of about 5.4 nm in diameter and 2.5 µm average length [27], [28]. PAO is a leading cause of serious congenital anomalies like urinary tract infections, bloodstream infections, surgical site infections, hospital-acquired pneumonia, respiratory morbidity and mortality in patients with cystic fibrosis (CF) [29], [30]. It is a frequent cause of exacerbations in the individuals with advanced chronic obstructive pulmonary disease [31]. Additionally, due to its frequent occurrence, nosocomial *P. aeruginosa* infections are often uncompromising with an enough mortality rate of approximately 50% for *P. aeruginosa* pneumonia patients [32]. As suggested above, *P. aeruginosa* infections are becoming more severe and a serious threat due to their continued emergence and spread of multi-drug resistant *P. aeruginosa* strains. Based on these factors, the therapeutic options are becoming increasingly limited. Thus, for the development of new drugs to target this highly infectious and medically important pathogen, our understanding of the pathogenesis of *P. aeruginosa* infections is critical.

Due to the recent recognition that proteins equate normal and aberrant cellular behavior by reinforcing defense against external pathogens, this study provides a basic insight into understanding the molecular mechanism of such control. It also signifies a novel drug target site to control the complications caused by *P. aeruginosa* during various infections. Thus, our study opens a revolutionary area of research by addressing the role of DYNLL1 in bacterial infections other than viral infections.

## Methods

### Data set

X-ray diffraction structure of human DYNLL1 (PDB code: 1CMI) was retreived through Protein Data Bank [33]. Experimentally verified binding motifs of DYNLL1 interaction partners (Table 1) were isolated through extensive litrature survey [1], [2], [7], [9], [15]. For Pilin, NMR-derived structure of *Pseudomonas aeruginosa* strain PAO (PDB ID: 1PAO) was used for docking simulations.

**Table 1:** Experimentally verified binding motifs (indicated by underline) of DYNLL1 interacting partners.

No.	Protein Name	Uniprot ID.	PDB ID.	Organism	Sequence
1	Adenain (ADE41)	P11826		Adenovirus	CITLVKSTQTV
2	AIBC1 (BCAS1)	O75363 Q3ZB98		Human, rat	KRMLDAQVQTD
3	ATMIN	O43313		Human	LESLDIETQTD
4	p54 (E183L)	Q4TWM2		ASF virus	VTTQNTASQTM
5	BimEL (BCL2L11)	O43521	1F95	Human	PMSCDKSTQTP
6	BS69 (ZMYND11)	Q15326		Human	PRMLHRSTQTT
7	DNMT3A	Q9Y6K1		Human	LVLKDLGIQVD
8	EML3	Q32P44	2XQQ	Human	PSLVSRGTQTE
9	Gephyrin (Gphn)	Q03555		Rat	KQTEDKGVQCE
10	Grinl1A (GCOM1)	P0CAP1		Human	TEVETREIGVG
11	E4	P06425		Papilloma virus	DHHQDKQTQTP
12	Hsc73 (Hspa8)	P63018		Rat	TTIPTKQTQTF
13	KID-1 (Znf354a)	Q02975		Rat	SHRTTKSTQTQ
14	MAP4	P27816		Human	SRSGSKSTQTV
15	Mark3	Q8VHF0		Rat	VVAYPKRSQTS
16	NEK9	Q8TD19		Human	VGMHSKGTQTA
17	nNOS (NOS1)	P29475	1F96, 1CMI	Human	AEMKDTGIQVD
18	NRF1	Q16656		Human	MEEHGVTQTE
19	p53BP1 (TP53BP1)	Q12888		Human	PSQNNIGIQTMETVVSAATQTI
20	PAK1	Q13153	3DVT	Human	TPTRDVATSPI
21	P protein	P15198		Rabies virus	RSSEDKSTQTT
22	P protein	O56780		Mokola Virus	KSTEDKSTQTP
23	RACK1 (GNB2L1)	P63244		Human	LGVCKYTVQDE
24	RASGRP3	Q8IV61		Human	RATTSQATQTE
25	U19	Q69501		HHV-7	TILVSRSTQTG LGHFTRSTQTS
26	UL9	P10193		HHV-1	GVQMAKSTQTF
27	Spice1	Q8K3I7		Mouse	QDVLRRTVQTR
28	Syntaphilin (Snph)	B5DF41		Rat	SCMQERAIQTD
29	TRPS1	Q9UHF7		Human	TEKVDRSTQDE
30	VP35	Q05127		Ebolavirus	PKTRNSQTQTD

### Identification of putative DYNLL1 binding motif containing proteins

**Figure 1:**
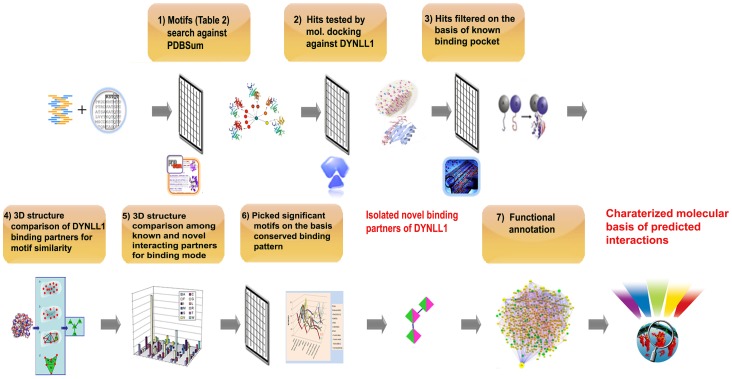
Schematic illustration of strategy. (**1**) Collected DYNLL1 binding motif sequences and PDBSum database was screened for these motifs. (**2**) Candidate proteins containing the DYNLL1 interaction motifs were picked (Table 2, bold hits). The selected proteins were tested by molecular docking against DYNLL1 to verify the hits which bind to DYNLL1 specific binding pocket. (**3**) Selected DYNLL1-interacting proteins on the basis of binding score values and common docking poses. (**4**) 3D structure comparison of DYNLL1 binding partners was carried out to examine the motif similarity pattern. (**5**) 3D structure comparison among known and novel DYNLL1 interacting proteins was conducted to inspect the structural conservation of putative DYNLL1 binding motifs and to evaluate the similarity of interacting mode. (**6**) Candidate proteins exhibiting highly conserved and identical binding pattern to the known one were selected. (**7**) Finally, annotated the possible pathways for the functional basis of novel interactions.

On the basis of specified consensus motif (K/R)XTQT for DYNLL1 binding partners, a homology search was performed against PDBsum database [34]. Linear motif search was accomplished by using the query sequences, particularly viral DYNLL1 binding motifs like KSTQT, RSTQT, KSIQI, KQTQT, SQTQT and TSTQT. The resulting hits with more than 75% sequence identity to the query sequence were collected. Later on, previously reported DYNLL1 interacting proteins (Table 2, unbold hits) were eliminated and the remaining hits (Table 2, bold hits) were selected for further analysis (Figure 1).

**Table 2:** DYNLL1 binding motif containing viral proteins.

Query Sequence (DYNLL1 binding motif)	Hits PDB ID.	% identity and sequence of hits
KSTQT	2P1K (C)	80%, KATQT
	**1RXZ(B)**	100.0%, KSTQA
	3GLW(Z)	80.0%, KQTQT
	**1PAN(A) 1PAO(A)**	100.0%, KSTQD
	**3K8Z(A)**	100.0%, KSTQT
	**3QRG(H)**	77.8%, KPTQT
RSTQT	**2GL7(F)**	80.0%, RSLQT
	**1V36(A) 3MHZ(A) 3Q8E(A) 3TEW(A) 3TEX(A) 3TEZ(A) 3TEY(A)**	87.5%, TDSQT
	**1F5J(A)**	75%, TFSQS
KSIQI	2WZL	100.0%, KSIQI
KQTQT	3GLW(Z)	100.0%, KQTQT
	3FM7(C)	100.0%, KQTQT
	**1MCA(A)**	100.0%, KQTQT
	**1DOM(A) 1DON(A)**	100.0%, KQTQT
	1CKR(A) 7HSC(A) 1Q2G(C)	100.0%, KQTQT
	1RY6(A)	100.0% KQTQT
	2PG1(I) 2PG1(J)	80.0%, KETQT
	3ER9(B) 2GA9(D) 2GAF(D) 3ER8(C) 3ERC(C) 3OWG(A)	100.0%, KQTQT
SQTQT	3FKE	100.0%, SQTQT
TSTQT	1INB	100.0%, TSTQT

Previously reported interacting partners are indicated in unbold, novel interacting partners (bold), while putative DYNLL1 interacting hits screened by docking simulations (bold and underlined).

To screen the candidate proteins which bind to DYNLL1 specific binding pocket, initial molecular docking analysis was performed using the short-listed data set (Table 2). Subsequently, on the basis of functional relevance to pathogenesis, Pilin was selected (1PAO) for detailed analysis.

### Molecular docking analysis of DYNLL1 and Pilin

Molecular docking analysis of DYNLL1 against Pilin was accomplished using five well known docking tools to obtain the best native conformation. These included AUTODOCK [35], PatchDock with refinement and re-scoring tool FireDock [36], [37], ZDOCK [38] and DOCK/PIERR [39] with refinement and re-scoring algorithm FiberDock [40] and CLUSPRO [41].

DYNLL1-Pilin semi flexible docking was carried out by a high performance OpenSuse linux system using AutoDock 4.0 [35]. Polar hydrogen atoms and KOLLMAN charges were assigned for DYNLL1 and number of active torsions in Pilin was set to 30 in order to perform docking simulation with rigid receptor (DYNLL1) and semi-flexible ligand (Pilin). The grid dimensions of 50×50×50 Å^3^ with a spacing 0.602 Å were set to generate the grid map. LGA was applied with the following parameters: docking consisted of 50 runs, initial population of 150 randomly placed individuals, 2.5×10^6^ energy evaluations, a maximum number of 27,000 generations, a mutation rate of 0.02 and a crossover rate of 0.80.

In case of PatchDock tool, the input parameters were the PDB coordinate files for DYNLL1 (1CMI) and Pilin (1PAO) with default parameters. Scoring function that considers both geometric fit and atomic desolvation energy [42] was used to evaluate each candidate transformation. Finally, Root Mean Square Deviation (RMSD) clustering was applied to the candidate solutions to discard the redundant solutions.

ZDOCK uses a grid-based representation and 3D Fast Fourier Transform (FFT) to effectively explore the rigid body search space of docking positions [43]. ZDOCK version 3.0.2 was used to study DYNLL1 and Pilin binding and FiberDock (Flexible Induced-fit Backbone Refinement in Molecular Docking) algorithm was used for the refinement and scoring of the ZDOCK results of DYNLL1-Pilin interaction. The model file (models_example.ent) was prepared and uploaded on FiberDock to improve the accuracy of the candidate solutions and to identify the near native solutions among the 10 best solutions of ZDOCK.

DOCK/PIERR docking algorithm is based on residue contact potential learnt using an SVM-based scoring function to minimize false positive rates and FFT based approximations of OPLS (Optimized Potentials for Liquid Simulations) vdw energy and electrostatic interactions. Side chain remodeling and energy minimization were used to perform refinement. Refined models were re-ranked using residue and atomic potentials for protein docking [44]. The top 10 refined models of DYNLL1-Pilin were retrieved from DOCK/PIERR server.

Another docking algorithm used in this study was CLUSPRO which works in three main steps. First, it runs PIPER, a rigid body docking program, based on a novel Fast Fourier Transform (FFT) docking method with pair wise potentials. Second, by using a clustering technique for the detection of near native conformations [45] and by eliminating some of the non-native clusters, the 1000 best energy conformations are clustered, and the 30 largest clusters are retained for refinement. Third, by short Monte Carlo simulations, stability of these clusters is analyzed, and by the medium-range optimization method SDU (Semi-Definite programming based Underestimation), the structures are refined. Input of both proteins in CLUSPRO server resulted in a file containing 4 categories of predicted models: (1) Balanced, (2) Electrostatic-favored, (3) Hydrophobic-favored and (4) VdW+Elec. Models in all the categories were ranked by cluster size. We focused on saturated clusters of best models in all categories.

### Comparative structural analysis of putative DYNLL1 binding motif

3D structures of DYNLL1 known interaction partners (BimEL (BCL2L11), DNMT3A, EML3, nNOS (NOS1), PAK1, RACK1 (GNB2L1) and Vaccinia polymerase) (Table 1) were obtained through PDB database [33] for performing structural comparisons among known and putative novel DYNLL1 binding motifs of candidate proteins. 3D structures of known interacting partners (Table 1) of DYNLL1 lacking experimentally determined structures were predicted by *ab initio* based method through I-TASSER server [46]. Subsequent visualization and superimposition of 3D structures were done using UCSF Chimera ver. 1.7.0 [47]. MolProbity [48], Verify 3D [49] and Errat [50] tools were used for model validation and WinCoot [51] was used for model refinement. The functionally important residues lying at the DYNLL1 binding site were screened through PDB database.

### Analysis of interaction dynamics

Molecular dynamic (MD) simulation of best docked Pilin-DYNLL1 complex was performed to evaluate the stability, folding, conformational changes and dynamic behavior of interacting proteins. All MD simulations were performed using Amber03 force field using GROMACS 4.5 package [52], running on high performance OpenSuse linux system. During MD simulations, all the systems were solvated using TIP4P [53] water model in a periodic box, followed by addition of Na^+^ and Cl^−^ counter ions to neutralize the systems. Before MD simulations, energy minimization (steepest descent algorithm for 500 steps) was performed by tolerance of 1000 kJ/mol Å^2^ to remove initial steric clashes. The energy minimized systems were treated for 1000 ps equilibration run under pressure and temperature conditions to relax the systems. Finally, MD simulations were run for 15 ns time scale under constant temperature (300 K) and pressure (1 atm). PME (Particle Mesh Ewald) algorithm was used in all calculations to dissect electrostatic interactions. Snapshots were collected throughout the MD simulations of each system and PDBs were generated for 1, 5, 10, 12 and 15 ns intervals to investigate the time-dependent behavior and stability of each system. UCSF Chimera ver. 1.7.0 [47], PyMol (http://www.pymol.org) and GROMACS tools were used to analyze the stability and behavior of each system.

## Results

### Analysis of Pilin specific motif

**Figure 2:**
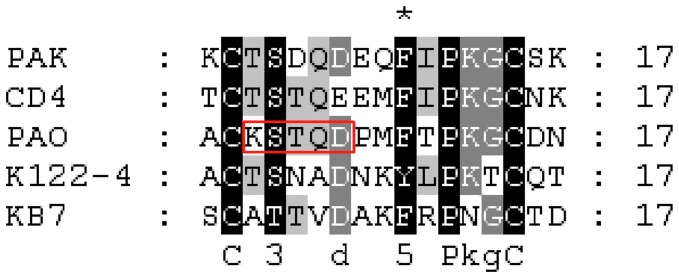
Conservation pattern of Pilin receptor binding domain. Pilin receptor binding domains (128-144 AA) of different strains of *P. aeruginosa* [60] are indicated and aligned. The Pilin protein of PAO strain contains a putative DYNLL1 binding motif which is highlighted by red rectangle. The black and dark gray colors show motif conservation pattern among four strains of *P. aeruginosa*, respectively.

Pilin (PDB ID: 1PAO) was found by screening the consensus DYNLL1 binding motif KSTQT against PDB database. It is a fimbrial protein of gram negative bacteria PAO, a strain of *P. aeruginosa* and encoded by the *pilA* gene. We identified that Pilin contains a homologous sequence (KSTQD) to DYNLL1 binding motif in its receptor binding domain (Figure 2). This motif is present in several DYNLL1-interacting proteins such as human adenovirus protease, BimEL, MAP4, P protein Rabies virus, P protein Mokola Virus, replication origin-binding protein (UL9) of human herpes virus 1 (HHV-1), human herpes simplex helicase and Amsacta moorei entomopox virus AMV179 ORF [2], [7].

Next, the conservation pattern of this putative DYNLL1 interaction motif was studied in Pilin receptor binding domain across different strains of *P. aeruginosa* (PAO, KB7, PAK, CD4 and K122-4). This analysis demonstrated that Ser131 and Asp134 residues are quite conserved, while Thr130 and Gln133 are less conserved among *P. aeruginosa* strains [54]. Interestingly, Thr at 130 position is replaced by Lys residue which is uniquely present in PAO strain (Figure 2).

### Comparative analysis of DYNLL1 and Pilin interaction by multiple docking protocols

**Figure 3:**
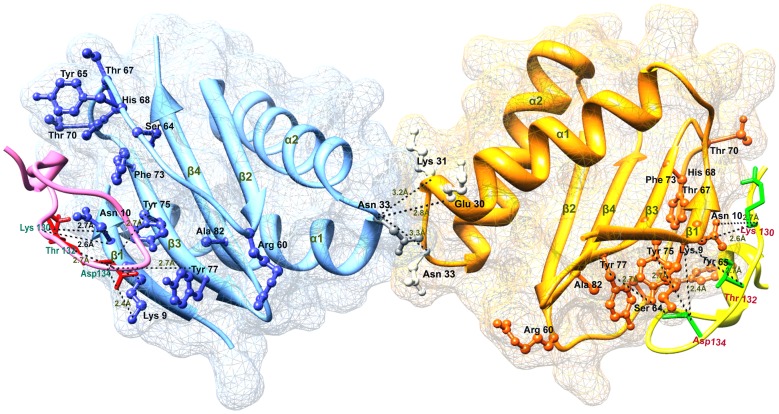
Residual contributions of DYNLL1 and Pilin explored through multiple docking protocols. The interacting residues of (A) DYNLL1 binding groove and (B) Pilin receptor binding domain are indicated. The residues marked in blue color are common among all the best docking poses, yellow and green colors indicate the residues which are common in 3 and 4 poses, respectively out of the 5 docking results. Pilin specific motif (KSTQD) is shown by black rectangle. Red rectangle highlights Lys130 residue which is only present in PAO strain. The docking/binding energy values for DYNLL1-Pilin obtained through individual docking procedures are: AUTODOCK, 0.9 kcal/mol; PatchDock, −28.31 kcal/mol; ZDOCK, −20.76 kcal/mol; DOCK/PIERR, −37.08 kcal/mol; CLUSPRO, −647.5 kcal/mol, respectively.

To analyze the reliability of interaction mode of Pilin and DYNLL1, five most modern docking tools including AUTODOCK, PatchDock, ZDOCK, DOCK/PIERR and CLUSPRO were used to accomplish the molecular dockings and to find the near native conformation of DYNLL1-Pilin complex. Out of the conformations generated by semi-flexible docking simulations of DYNLL1 and Pilin by AUTODOCK, potential DYNLL1-Pilin clusters were ranked on the basis of lowest energy representation and RMSD values, using the default threshold (2.0 Å). DYNLL1-Pilin complex having the minimal energy of 0.9 kcal/mol clearly depicted the Pilin binding to the reported binding pocket of the DYNLL1 (Figure 3). Through PatchDock analysis, the ten best solutions were selected for further refinement and rescoring analysis by FireDock (**Fast Interaction REfinement in molecular DOCKing**) algorithm. The desolvation energy value for the best docking model was −8.43 kcal/mol (Figure 3), which revealed a high feasibility of this binding [55]. ZDOCK generated 2000 conformations of DYNLL1-Pilin complex and the top 500 models of complex were visualized in JMOL [56]. Interestingly, almost all models exhibited a similar pattern of Pilin interaction with the reported DYNLL1 binding pocket [7], [57], [58]. Desolvation energy (−9.40 kcal/mol) and a global free energy (−20.76 kcal/mol) (Figure 3) profile indicated a high interaction probability of DYNLL1 and Pilin. DOCK/PIERR output of DYNLL1-Pilin interactions were refined and re-scored using FiberDock. The best output model with a high desolvation (−11.03 kcal/mol) and global energy (−37.08 kcal/mol) was studied (Figure 3), which further support validated the Pilin binding with at the same DYNLL1 binding pocket. Altogether, out of the top 10 models, 7 conformations supported Pilin complementary with DYNLL1 binding pocket. To reliably generate high-quality structures of DYNLL1-Pilin complexes, another highly effective multistage docking approach CLUSPRO was used by employing a simplified energy function and limited flexibility to discover regions of interest. The sizes and lowest energy values for largest clusters of balanced were 272 and −647.5, electrostatic-favored were 287 and −691.1, hydrophobic-favored were, 303 and −750.1 and VdW+Elec were 310 and −146.4, respectively. The interacting residues of the representative models out of the largest clusters were analyzed by LIGPLOT [59].

**Figure 4:**
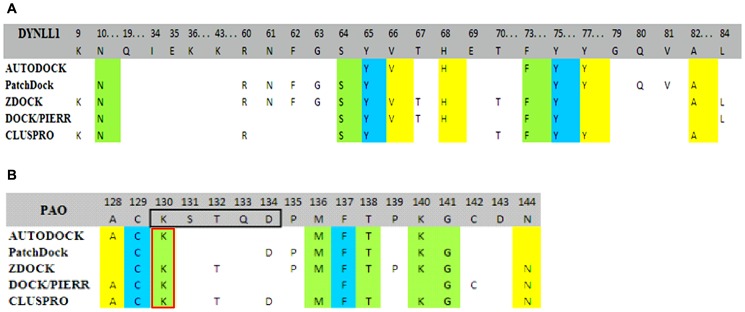
Visualization of the best docked DYNLL1-Pilin complex in dimer form. Blue and orange ribbons indicate individual DYNLL1 monomers connected by H-bonds as indicated. Pilin is shown in ribbon form in pink and yellow colors. Interacting residues are shown in atomic representations and H-bonds are shown by dotted black lines. Pilin specific residues (Lys130, Thr132 and Asp134) are involved in hydrogen bonding.

The best DYNLL1-Pilin complexes predicted by the mentioned algorithms presented Pilin binding in the similar binding pocket of DYNLL1 (Figure 4), which substantiated sufficient evidence of their interaction (Figure S1–2).

### Comparative binding analysis of Pilin to other DYNLL1 known targets

Binding pocket details for DYNLL1 were gathered through PDB database by studying PDB entries including 1F95 (bim peptide and dynein light chain 8 (dlc8) complex), 2XQQ (peptide (AC-SRGTQTE) in complex with human dynein light chain), 1CMI (dimer of the human DYNLL1 with a bound peptide) and 1F96 (nNos peptide and dynein light chain 8 (dlc8)) complex. In these complexes, we observed Thr70, Gly79, Glu69, Tyr77, Tyr75, Gln80, Gly63, Ala82, Phe62, Asn61, Thr67, Arg60, Tyr65, Val66, Ser64, His68, Phe73, Leu84, Lys36, Lys43, Glu35, Ile34, Gln19, Lys9, Asn10 and Val81 residues of DYNLL1 involved in binding with mentioned ligands [7], [57], [58].

**Figure 5:**
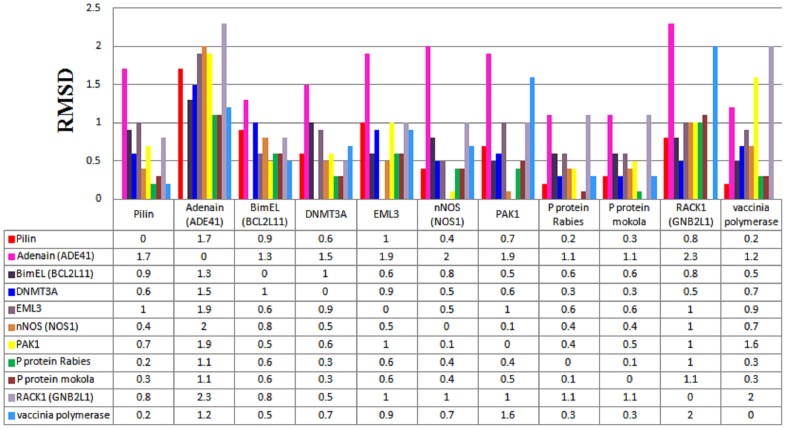
Structural comparisons of all possible combinations of binding motifs. Individual RMSD values are plotted against the corresponding superposed motifs to all the indicated proteins including Pilin (KSTQD), Adenain (KSTQT), BimEL (KSTQT), DNMT3A (LGIQV), EML3 (RGTQT), nNOS (TGIQV), PAK1 (DVATS), Rack1 (YTVQD), P protein Mokola virus (KSTQT), P protein Rabies virus (KSTQT) and Vaccinia polymerase (KQTQT). The RMSD analysis shows that Pilin specific motif contains a close structural similarity to the validated DYNLL1 binding motifs.

Next, to validate the biological significance of putative Pilin-DYNLL1 binding motif, 3D structural comparisons of previously reported DYNLL1 binding motifs for BimEL, Adenain, DNMT3A, EML3, nNOS, PAK1, Rack1, Vaccinia polymerase P protein of Mokola and P protein Rabies viruses [2], [6], [7] were performed. These structures were superimposed for comparing the binding motif conservation structurally to further narrow down the interaction crosstalk. As indicated in Figure 5, Pilin peptide exhibited close structural similarity with Vaccinia polymerase and P protein Rabies by sharing an RMSD (Root mean square deviation) value of 0.2 Å. Similarly, superimposition of Pilin and P protein Mokola resulted in RMSD value of 0.3 Å (Figure 5). These data indicated that Pilin peptide (KSTQD) attains a similar binding pattern to that of Vaccinia polymerase (KQTQT), P protein Rabies (KSTQT) and P protein Mokola (KSTQT) viruses by sharing a common 3D architecture (Figure S3).

### MD simulation analysis

**Figure 6:**
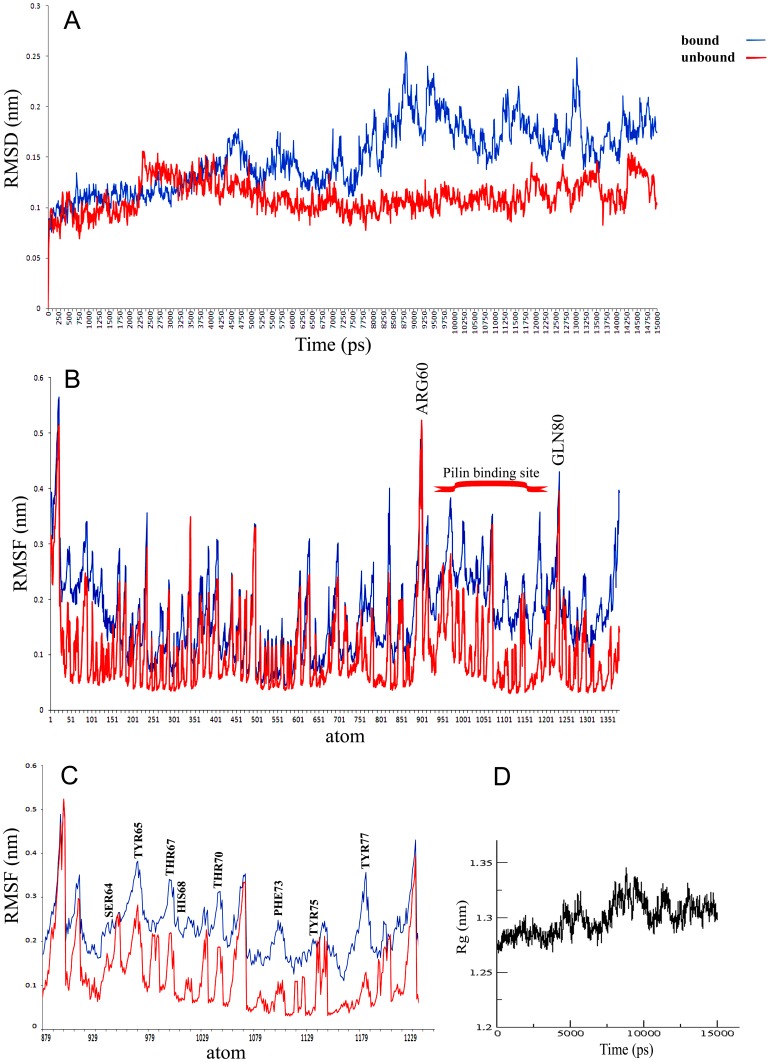
Stability and residue fluctuations of 15(DYNLL1-Pilin). (A) RMSD plot of Cα atoms computed through bound DYNLL1-Pilin complex (blue) and unbound structure (red). (B) Comparison of the bound (blue) and unbound (red) structures quantified by their backbone root-mean-square-fluctuations (RMSF). The pilin binding site is indicated. (C) Comparative RMSF plot for DYNLL1-loop region between Arg60 and Gln80. (D) Radius of gyration (Rg) plot for DYNLL1-Pilin complex.

**Figure 7:**
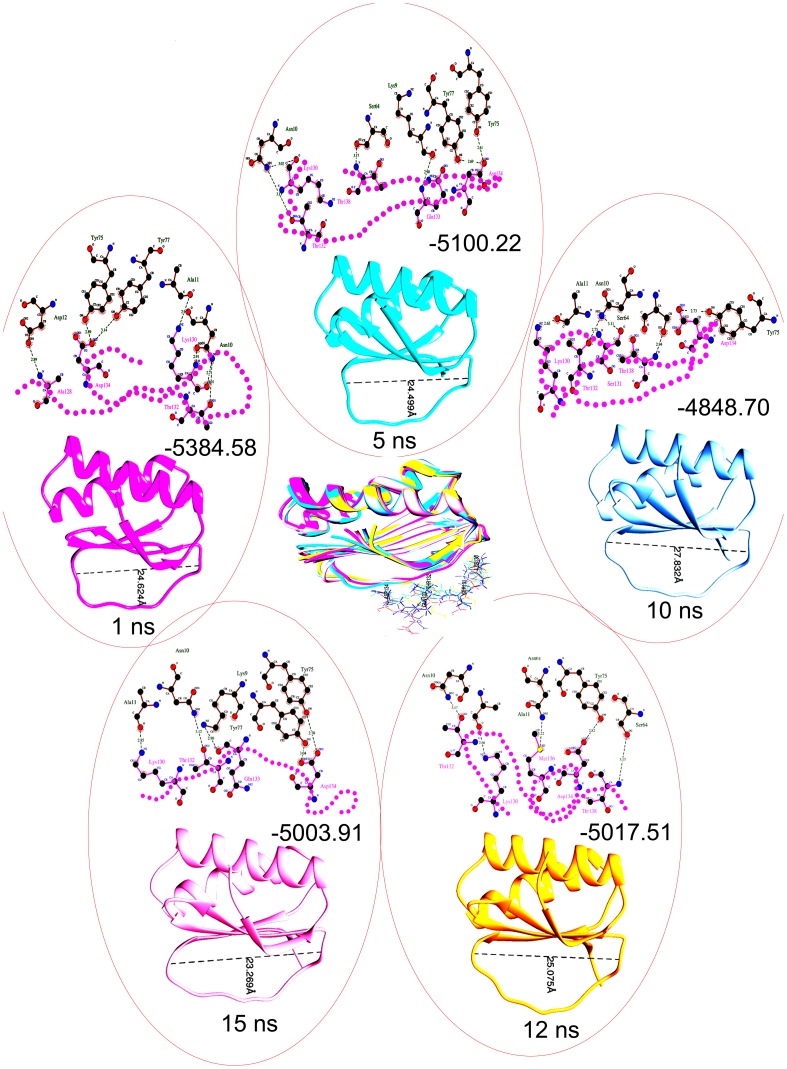
Structural and conformational adjustments of DYNLL1-Pilin system at different time scales. Snapshots were collected throughout the MD simulations of each system and PDBs were generated for 1, 5, 10, 12 and 15-dependent behavior and stability of each system. PDB structures were visualized in UCSF Chimera 1.7.0. 2D plots were generated through DIMPLOT and residues involved hydrogen bonding (indicated by black dotted lines) were monitored. The Pilin specific residues involved in hydrogen bonding are indicated by joining pink dotted circles. The observed DYNLL1-loop measurements were 24.62 Å, 24.49 Å, 27.83 Å, 25.07 Å and 23.36 Å, respectively. The binding energy values at indicated time scales were −5384.58 kJ/mol, −5100.22 kJ/mol, −4848.70 kJ/mol, −5017.51 kJ/mol and 5003.91 kJ/mol, respectively. Superimposed structures at the indicated time intervals for DYNLL1 are indicated by pink (1 ns), cyan (5 ns), blue (10 ns), pink (12 ns) and yellow (15 ns) colors, respectively.

The stability of secondary structure elements and conformational changes of simulated complexes were assessed by plotting root mean square deviation (RMSD), root mean square fluctuation (RMSF) and radius of gyration (Rg) values, obtained throughout the trajectory. Backbone RMSD scores observed over a period of 15 ns for DYNLL1 remained stable until 8 ns (1–1.5 Å), however RMSD behavior showed a marked increase during 9–13.5 ns time period, indicating more structural rearragements (Figure 6A). Subsequent analysis of root mean square fluctuation (RMSF) by residue indicated fluctuations between Arg60 (up to 5Å) and Gln80 (up to 4.5Å) (Figure 6B). To our surprise, DYNLL1-loop region amino acids involved in Pilin binding (Ser64, Tyr65, Tyr67, His68, Thr70, Phe73, Tyr75, Tyr77) exhibited more fluctuations (2–4 Å) (Figure 6C). Radius of gyration (Rg) is a simple measure of stability and firmness of the system and tends to change over time due to protein folded-unfolded states [60]. The calculated 2D plot for mean Rg for the system was consistent with RMSD profile (Figure 6D). In agreement to RMSD data, Rg profile for Pilin-DYNLL1 was elevated during 9–13.5 ns, indicating conformational changes due to structural flexibilty upon binding. An overall trend of RMSD (below 2.5Å) for bound and unbound systems indicated that systems were well equlibrated and validated the stability of complex during simulations. Next, individual simulated complexes were monitored by generating trajectories for 1 ns, 5 ns, 10 ns, 12 ns and 15 ns time intervals to investigate the time-dependent dynamics of individual amino acids for Pilin-DYNLL1. It was observed that Pilin exhibited a quite stable binding pattern with DYNLL1 by sharing residues located at the DYNLL1 binding motif (KSTQD) with varying binding affinities (Figure 7). As DYNLL1-loop attained more conformational changes upon Pilin binding, its orientations were deeply analysed by measuring its length at mentioned time scales. In analysed trajectories, the observed sizes of loops were 24.62 Å, 24.49 Å, 27.83 Å, 25.07 Å and 23.36 Å, respectively (Figure 7). The binding energy values at these time scales were −5384.58 kcal/mol, −5100.22 kcal/mol, −4848.70 kcal/mol, −5017.51 kcal/mol and −5003.91 kcal/mol, respectively. Moreover, MD simulations substantiated that DYNLL1 specific Lys9, Asn10, Ala11, Ser64, Tyr75 and Tyr77 residues were involved in hydrogen bonding with Ala128, Lys130, Thr132, Gln133, Asp134, Met136 and Thr138 residues of Pilin (Figure 7).

## Discussion

Here, we employed a homology search using DYNLL1 specific consensus binding motif (K/R)XTQT and elucidated Pilin-DYNLL1 interaction through comparative docking simulations. Interestingly, MD simulation data and docking clusters revealed the involvement of Pilin specific Lys130 residue in DYNLL1 binding. This residue is uniquely present only in PAO strain of *Pseudomonas aeruginosa* thus raising the possibility that KSTQD binding motif of Pilin might be evolved due to substitution of Thr into Lys. Through MD studies, Rg and RMSD plots of the protein backbone showed convergence of simulated complex below 2.5 Å, indicating that all the systems were well equilibrated and stable throughout simulations. An increasing RMSD trend at 9 ns time scale is the indicative of mobility with in the binding pocket and depicted the structural flexibilty induced due to binding of Pilin. A more detailed analysis of trajectories at different time intervals explained a a positive role of DYNLL1-loop flexibilty in the stablized binding as shown by RMSF measurements (Figure 6). Particularly, the amino acids, namely, Ser64, Tyr65, Thr67, His68, Thr70, Phe73, Tyr75 and Tyr77 of the DYNLL1-loop region have shown more fluctuations upon Pilin binding, compared to the unbound form. This differential affinity of Pilin peptide towards DYNLL1-loop seems to be correlated with the subtle changes in the loop conformation, as widening of loop (27.83 Å) at 10 ns time interval resulted in a more stronger binding (−4848.70 kcal/mol). It is possible that this narrowing and widening of loop might adjust the KSTQD motif to establish interactions with the Lys9, Asn10 and Ala11 residues of DYNLL1-β1, closely located to the loop region. Interestingly, Pilin association to DYNLL1 resulted in conformational rearrangements of β1 by extending it slightly (Figure 7). These time-dependent binding characteristics can also be observed in focused Rg plot, where an increase of gyration rate was observed during 9–10 ns time interval (Figure 6). Overall, these results confirmed that intermolecular crosstalk of Pilin and DYNLL1 is stablized due to structural rearrangements, consequently leading to a more pronounced binding.

To authenticate the DYNLL1-Pilin interaction, we characterized the known binding partners of DYNLL1 having similar binding pattern, where structural overlay of Pilin peptide showed a striking similarity to that of Vaccinia polymerase and P protein Rabies. Given the association of only 12% DYNLL1 in dynein complex [61], we hypothesized implication of the free form of DYNLL1 in transportation independent processes by inducing an uncontrolled inflammatory response during lung chronic infection of *P. aeruginosa*, which causes most of the morbidity and mortality in CF patients [62], [63]. This process of increased rate of inflammation in response to Pilin stimulus [64] draw our attention to the preferable involvement of NF-κβ pathway in *P. aeruginosa* infection process. In mammalian cells, most common subunits of NF-κβ are RelA (p65) and p50, which lie in heterodimer form. Under basal conditions, cytosolic NF-κβ remains inactive due to the inhibitor protein Iκβα [65]. However, in response to a variety of stimuli including TNFα, interleukin (IL)-1β, lipopolysaccharide (LPS), pili [64] and flagellin [66], Iκβα gets phosphorylated by IKK (cytokine-activated IkappaB kinase) thereby resulting in its ubiquitination and degradation. Due to these events, nuclear localization signal (NLS) of NF-κβ is exposed which results in its translocation to the nucleus, where it regulates the transcription of multiple target genes [65].

**Figure 8:**
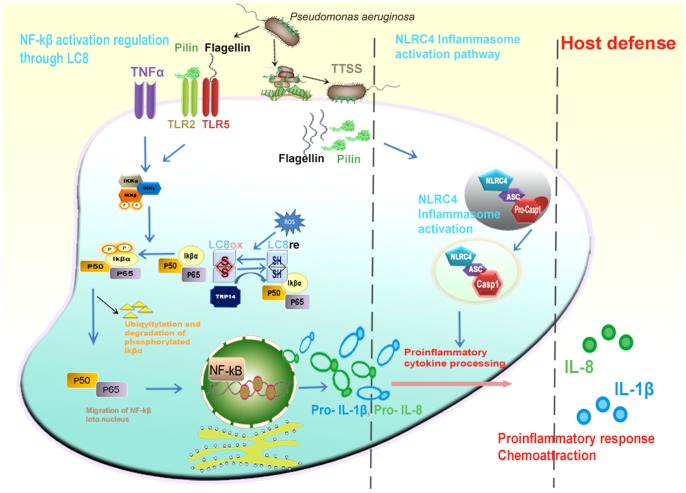
Roles of DYNLL1 and Pilin in host defense mechanism. Model of normal cell host defense mechanism. Pilin and flagellin trigger inflammasome activity in NLRC4 inflammasome activation pathway. DYNLL1/LC8 regulates NF-κβ activation by inhibiting the phosphorylation of Iκβα by Iκβ kinase.

**Figure 9:**
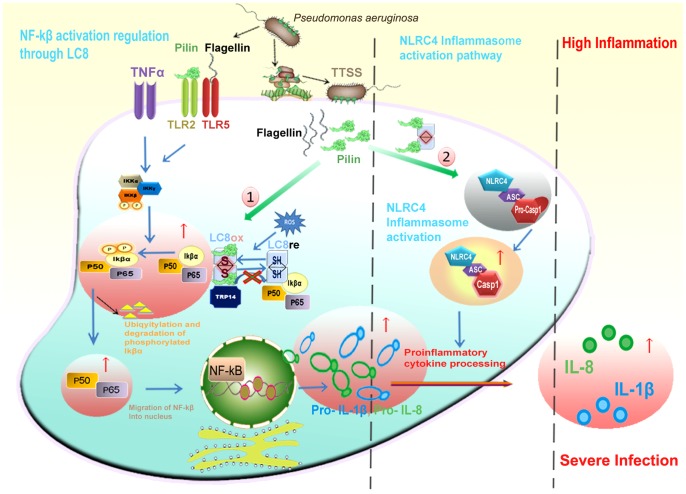
Role of DYNLL1-Pilin interaction during chronic inflammation. Model of *P. aeruginosa* infected cell pathway. (1) DYNLL1/LC8-Pilin interaction interrupts the reduction mechanism of TRP14 by maintaining DYNLL1 in oxidized homodimeric form which is unable to bind with Iκβα. Iκβα is instantly degraded due to continuous phosphorylation by Iκβ kinase, resulting in up regulation of NF-κβ pathway. (2) Pilin hijacks DYNLL1 to obtain compartmental specificity and inflammasome activation.

NF-κβ activation triggers inflammatory responses by releasing interleukins IL-1β and IL-8 cytokines [63], [65]. Reasonably, there have been reported several different but interconnected concepts involving the stimuli like TNFα, pili, IL-1β and lipopolysaccharide for the generation of reactive oxygen species to oxidize DYNLL1 [65]. The oxidized form of DYNLL1 homodimer is stabilized by the formation of reversible disulfide bond and attains a conformational change that causes its dissociation from the Iκβα, allowing Iκβα phosphorylation and NF-κβ activation (Figure 8). Possibly, Pilin interaction with DYNLL1 may lead to self-aggregation and hijacking of DYNLL1 to transport Pilin into the specific cellular compartments for inflammasome activation, causing DYNLL1 deficient environment. This may interrupt a 14-kDa human thioredoxin (Trx)-related protein TRP14 (disulfide reductase) to carry out the reduction process of DYNLL1. This unique role of TRP14 protein is not unprecedented as it has been reported to inhibit tumor necrosis factor α (TNFα) induced NF-κβ activation by maintaining DYNLL1 in a reduced state, where DYNLL1 depletion causes an increased activation of NF-κβ and overproduction of p65 [65] (Figure 9). As oxidized DYNLL1 is unable to bind with Iκβα, subsequent unchecked and prolonged phosphorylation of Iκβα by Iκβ kinase and NF-κβ activation may provide a possible clue of excess inflammatory response caused by *P. aeruginosa* infection in the CF cells. Thus as a fair assessment, major alterations in DYNLL1 cellular distribution and binding perturbations might be coupled dynamically to the subtle regulatory mechanisms during infections.

Initially, it has been observed that exaggerated cytokine responses of CF airway epithelial cells are due to the increased adherence of PAO to the CF cells. However, later on, no connection was observed between the high inflammatory response and *P. aeruginosa* adherence [63], which suggested the presence of an alternative mechanism for *P. aeruginosa* infection. Our proposed model serves as an alternative hypothesis, where type IV pili are delivered in a dependable process based on the bacterial TTSS (type III secretion system) [67]. TTSS is a mechanism whereby virulence factors including Pilin are introduced into the host cell by gram negative bacteria [68], which may contribute in host defense response through the activation of inflammasome. Pilin plays a crucial role in the host defense mechanism by inflammasome activation and production of IL-1β, which is a pivotal inflammatory cytokine and mediates the phagocyte activation, T-cell polarization and antibody production [69], [70]. Although, activation of inflammasome via Pilin (when injected by TTSS mechanism) is dependent on the NLRC4 (NOD-like receptor family, CARD domain-containing protein 4) and ASC (Apoptosis-associated speck-like protein containing a C-terminal caspase recruitment domain) components; however, upon introduction into cells by liposomal delivery, activation of caspase-1 (CASP-1) did not require NLRC4 and ASC proteins [67]. These data indicated that the sensors implicated in the identification of cytoplasmic danger signals like Pilin or viral DNA are compartmentalized, and that the diverse inflammasome components may be utilized in various compartments within cell.

Overall, our model provided the basis for understanding the mechanism of PAO infection through the possible involvement of Pilin by mediating an excess inflammatory response either by forcing DYNLL1 in oxidized state due to the interruption of TRP14 reduction mechanism or restricting DYNLL1 compartmentalization during inflammasome activation. Nevertheless, several biological and molecular mechanisms are possibly involved in mediating the complicated relationship between *P. aeruginosa* infections and exaggerated inflammation responses. Our model devised an excellent “infection hypothesis” by assessing a novel role of DYNLL1 in *P. aeruginosa* pathogenesis, and emphasized the need to further investigate the role of DYNLL1 during bacterial virulence.

## Supporting Information

Figure S1 **Surface view of common binding groove.** DYNLL1 (yellow) identified through comparative docking strategy. Pilin specific residues are indicated in the form of purple wires. Binding pocket residues of DYNLL1 are labeled in black color and distances are shown by solid lines (black).
(TIF)

Figure S2 **Interaction residue analysis for all complexes.** (A) AUTODOCK, (B) CLUSPRO, (C) DOCK/PIERR, (D) PatchDock and (E) ZDOCK.
(TIF)

Figure S3 **Superimposition of DYNLL1 binding motifs.** (A) Vaccinia virus polymerase (KQTQT) and Pilin (KSTQD), (B) P protein of Mokola virus (KSTQT) and Pilin (KSTQD), (C) P protein Rabies virus (KSTQT) and Pilin (KSTQD). Pink, blue, green and black colors represent the binding motifs of Pilin, Vaccinia virus polymerase, P protein of Mokola virus and P protein of Rabies virus, respectively.
(TIF)
