# Correction: Comparative Molecular Docking Analysis of Cytoplasmic Dynein Light Chain DYNLL1 with Pilin to Explore the Molecular Mechanism of Pathogenesis Caused by *Pseudomonas aeruginosa* PAO

**DOI:** 10.1371/annotation/2ee0288f-bbc7-4fb8-950b-4c504174f843

**Published:** 2013-11-15

**Authors:** Samina Kausar, Muhammad Asif, Nousheen Bibi, Sajid Rashid

The titles and legends for Figures 3 and 4 were incorrectly switched. The title and legend currently appearing with Figure 3 belong with Figure 4, and the title and legend currently appearing with Figure 4 belong with Figure 3. The images themselves are in correct order. 

