# Supplementary figures and images for: Comparative Molecular Docking Analysis of Cytoplasmic Dynein Light Chain DYNLL1 with Pilin to Explore the Molecular Mechanism of Pathogenesis Caused by Pseudomonas aeruginosa PAO
Source: PLoS One. 2013 Oct 3;8(10):e76730. doi: 10.1371/journal.pone.0076730 (PMC3789673; doi:10.1371/journal.pone.0076730)

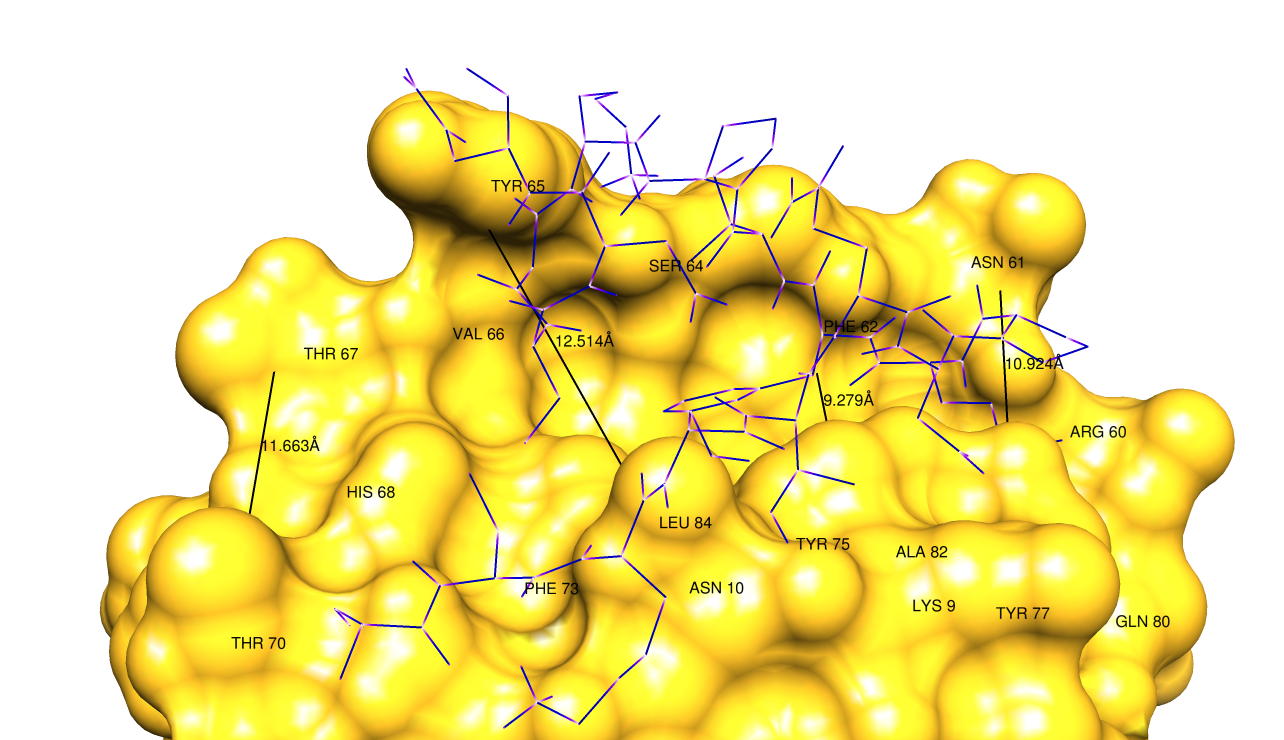

Supplement: Figure S1 — Surface view of common binding groove. DYNLL1 (yellow) identified through comparative docking strategy. Pilin specific residues are indicated in the form of purple wires. Binding pocket residues of DYNLL1 are labeled in black color and distances are shown by solid lines (black). (TIF) [file pone.0076730.s001.tif]

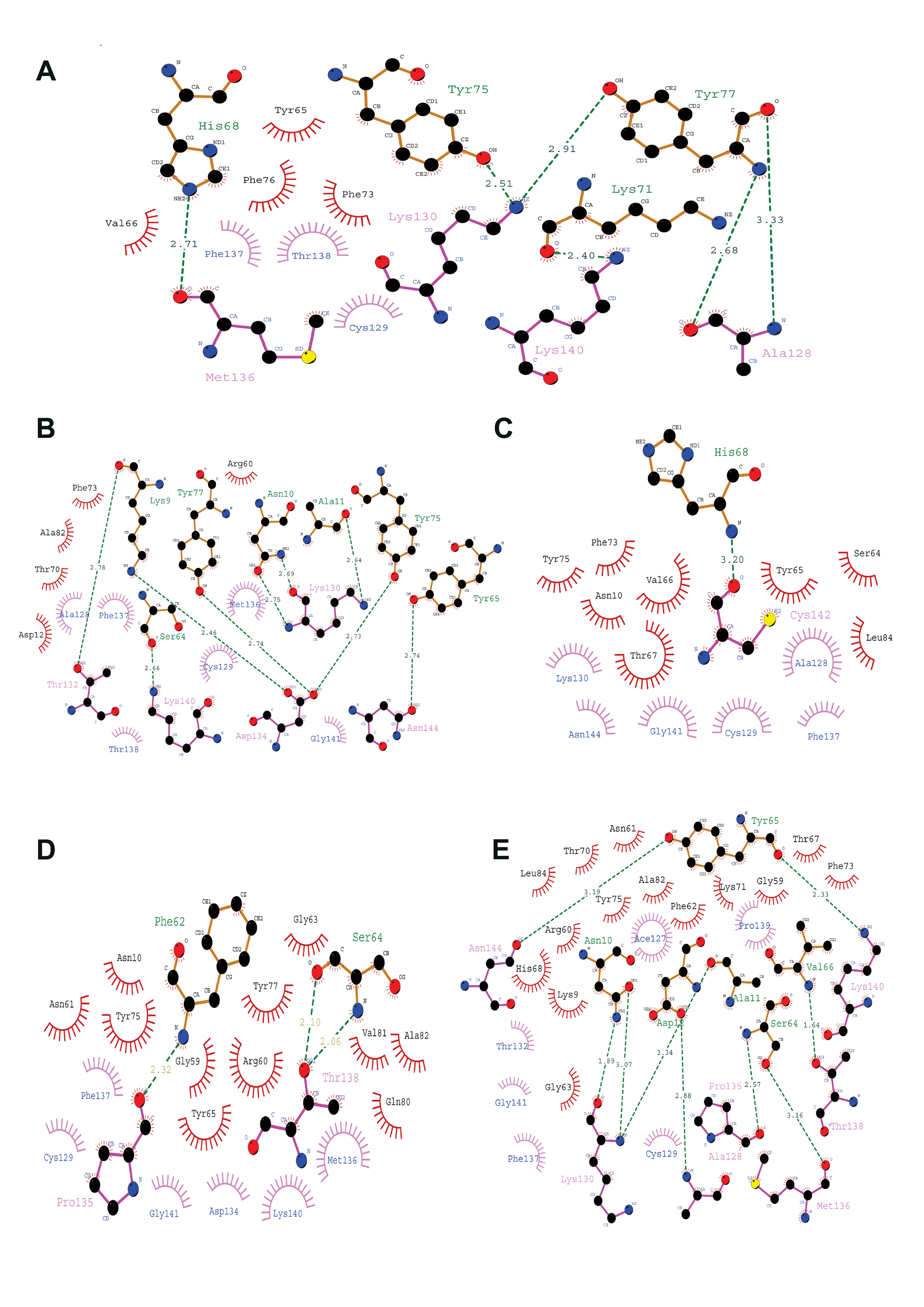

Supplement: Figure S2 — Interaction residue analysis for all complexes. (A) AUTODOCK, (B) CLUSPRO, (C) DOCK/PIERR, (D) PatchDock and (E) ZDOCK. (TIF) [file pone.0076730.s002.tif]

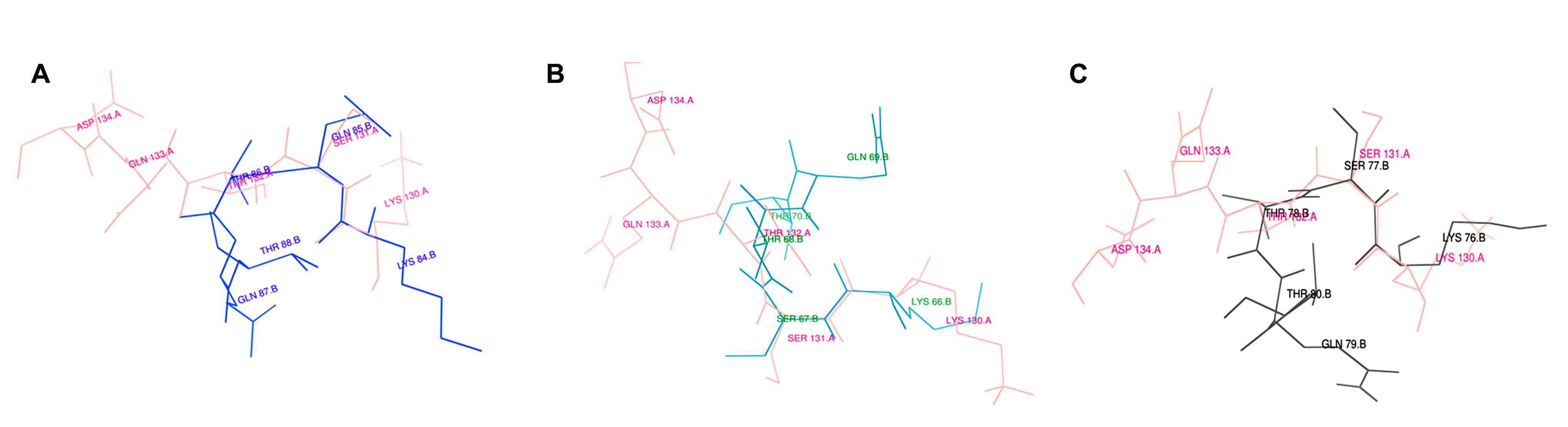

Supplement: Figure S3 — Superimposition of DYNLL1 binding motifs. (A) Vaccinia virus polymerase (KQTQT) and Pilin (KSTQD), (B) P protein of Mokola virus (KSTQT) and Pilin (KSTQD), (C) P protein Rabies virus (KSTQT) and Pilin (KSTQD). Pink, blue, green and black colors represent the binding motifs of Pilin, Vaccinia virus polymerase, P protein of Mokola virus and P protein of Rabies virus, respectively. (TIF) [file pone.0076730.s003.tif]
